# Reciprocal relationship between boredom proneness and problematic social media use among Chinese adolescents: a random intercept cross-lagged panel model

**DOI:** 10.3389/fpsyt.2026.1925655

**Published:** 2026-09-07

**Authors:** Su-Ting Chen, Fu-Ming Xu

**Affiliations:** 1 School of Education Science, Nanning Normal University, Nanning, Guangxi, China; 2 School of Psychology, Yunnan Normal University, Kunming, Yunnan, China

**Keywords:** adolescents, boredom proneness, longitudinal study, problematic social media use, RI-CLPM

## Abstract

**Introduction:**

Boredom proneness is a relatively stable dispositional vulnerability associated with adolescents’ problematic social media use (PSMU). However, it remains unclear whether this association reflects stable between-person differences, within-person temporal dynamics, or both. This study examined the reciprocal longitudinal associations between boredom proneness and PSMU among Chinese adolescents.

**Methods:**

Four-wave data were collected from 2020 to 2022 from 2,163 adolescents aged 12–19 years at baseline (mean age = 14.54 years, SD = 1.49; 47.4% female) attending four secondary schools in Guangzhou, China. A random-intercept cross-lagged panel model (RI-CLPM) was used to separate stable between-person differences from occasion-specific within-person deviations while controlling for gender, age, paternal education, and maternal education. Ethical approval and written informed consent were obtained.

**Results:**

At the between-person level, the stable components of boredom proneness and PSMU were positively associated (B = 0.195, p < 0.001). At the within-person level, an asymmetric prospective pattern emerged. Higher-than-expected boredom-proneness scores predicted higher-than-expected PSMU only from T1 to T2 (B = 0.073, p < 0.05), whereas higher-than-expected PSMU consistently predicted higher-than-expected boredom-proneness scores across all three adjacent intervals (B = 0.074, 0.108, and 0.103; all p < 0.05). Both constructs also showed significant autoregressive effects.

**Discussion:**

The association between boredom proneness and PSMU comprised both stable between-person co-occurrence and asymmetric within-person temporal dynamics. PSMU consistently preceded subsequent elevations in boredom-proneness scores, whereas the reverse pathway was interval-specific. These findings do not establish causality or indicate that PSMU causes momentary state boredom or enduring personality change. They may help inform time-sensitive prevention and support strategies by distinguishing stable vulnerability from occasion-specific variation. Because all waves occurred during the COVID-19 pandemic, replication in post-pandemic cohorts is needed.

## Introduction

Adolescence is a developmentally sensitive period marked by substantial cognitive, emotional, and social change. The present sample, aged 12–19 years, spans much of the transition from early to late adolescence, during which sensitivity to peer feedback and social reward is heightened and self-regulatory capacities continue to mature (1, 2). Boredom is also common during adolescence and is associated with sensation seeking and maladaptive behavioral outcomes (3). Because social media provides immediate novelty, rapid feedback, and continuous peer interaction, it may become an accessible means of coping with understimulation, making adolescence a critical period for examining boredom-related maladaptive social media engagement.

Problematic social media use (PSMU) is conceptualized as a maladaptive but subclinical pattern of social media engagement characterized by impaired control, excessive preoccupation, and persistent use despite negative consequences (4, 5). Among adolescents, PSMU has been associated with poorer sleep and psychosocial maladjustment, including emotional distress, poorer academic performance, and impaired social functioning (6–8). These outcomes highlight the importance of identifying dispositional vulnerabilities and reciprocal processes associated with PSMU.

China is not merely the sampling location but also a context in which the association between boredom proneness and PSMU may be shaped by a particular platform–family–school ecology. Chinese adolescents use multifunctional platforms such as WeChat and QQ alongside algorithm-driven short-video platforms such as Douyin, while study-related stress, difficulties in family and school relationships, parental phubbing, and parental mediation have been linked to problematic digital engagement among Chinese youth (9–11). This ecology may shape how adolescents use social media to cope with understimulation and how problematic engagement persists over time. However, because no cross-cultural comparison was conducted, the present study characterizes these associations within the Chinese context rather than testing whether they are uniquely Chinese.

Prior research has examined several partially overlapping classes of factors associated with PSMU, including broad personality dispositions (12, 13), psychological characteristics and use-related motives (14), and negative emotional experiences (14, 15). These classes operate at different conceptual levels rather than representing mutually exclusive categories. Psychological and motivational factors generally refer to proximal needs, self-evaluations, or reasons for engaging with social media, whereas negative emotional factors capture affective experiences or symptoms that may prompt compensatory use. Boredom proneness, by contrast, is conceptualized as a personality-related dispositional factor rather than a cognitive-motivational factor or a current emotional state. It reflects a relatively stable, cross-situational susceptibility to experiencing boredom, including difficulties in maintaining attention, interest, and adequate stimulation (16, 17). Relative stability does not imply that repeated boredom proneness scores remain identical across occasions; adolescents may show occasion-specific deviations from their own expected levels.

The present study focuses on boredom proneness for three reasons. First, compared with broad personality dimensions such as the Big Five, boredom proneness is more theoretically proximal to the affordances of social media. The rapid novelty, immediate feedback, and continuous interaction provided by social media may be particularly appealing to individuals who have difficulty maintaining stimulation and engagement (18, 19). Second, although boredom proneness is associated with negative affect and motivational difficulties, it is neither a current emotional state nor a specific motive for social media use. Instead, it represents a dispositional vulnerability that may shape how adolescents respond to situations perceived as insufficiently stimulating. Third, boredom is highly prevalent and developmentally salient during adolescence, yet longitudinal research has rarely examined whether boredom proneness and PSMU predict one another over time (3, 20). Focusing on boredom proneness therefore allows the present study to move beyond broad personality taxonomies and more proximal affective or motivational correlates, while using the RI-CLPM to distinguish stable between-person differences from within-person fluctuations (21). Importantly, focusing on boredom proneness does not imply that it is the sole or strongest determinant of PSMU. Rather, the present study examines one theoretically proximal and developmentally salient dispositional factor whose reciprocal longitudinal association with PSMU remains underexplored. The study was not designed to compare boredom proneness with all other individual, family, peer, or platform-related determinants of PSMU.

### Boredom proneness as a vulnerability factor for PSMU

Boredom is highly prevalent among contemporary adolescents (3). Cross-sectional evidence suggests that approximately 91%–98% of adolescents experience varying levels of boredom during their developmental years (22). In addition, longitudinal research has documented a notable increase in boredom tendencies among adolescents over time, with this upward trend being particularly pronounced among female adolescents (20). Scholars have suggested that such patterns may be partially attributable to highly digitalized, repetitive, and monotonous social environments, which are known to intensify experiences of boredom (23).

From a structural perspective, boredom is commonly conceptualized as comprising two distinct forms: transient state boredom and stable trait boredom, often referred to as boredom proneness. Trait boredom reflects a dispositional tendency characterized by chronically low arousal, difficulties in sustained attention, and diminished motivation in response to both external stimuli and internal cognitive activities (17). As a relatively stable personality characteristic, boredom proneness has been shown to play a critical role in shaping media-related behaviors and vulnerability to addictive patterns. Empirical studies consistently demonstrate that individuals with high levels of boredom proneness are more likely to engage excessively in smartphone use (24), develop internet addiction (18), participate in problematic online gaming (25), and exhibit PSMU (26). Accordingly, boredom proneness has been increasingly recognized as a specific dispositional vulnerability relevant to PSMU.

PSMU may serve regulatory functions by allowing adolescents to temporarily escape unpleasant internal states or obtain immediate psychological rewards through online interaction. From this perspective, PSMU is theoretically relevant to boredom proneness because adolescents susceptible to boredom may be more likely to rely on social media as an easily accessible source of stimulation, distraction, and emotional regulation.

### The reciprocal longitudinal association between boredom proneness and PSMU

To integrate the relevant perspectives, the present study adopts compensatory internet use theory as the overarching framework (27), while optimal-arousal accounts specify why boredom-prone adolescents may seek external stimulation (28, 29). Adolescents high in boredom proneness are more likely to experience insufficient stimulation and difficulty sustaining attention and engagement (17, 30). Because social media provides rapidly changing content, immediate feedback, and continuous social interaction, it offers a readily accessible means of compensating for understimulation (18). Temporary relief and immediate rewards may reinforce repeated use, increasing the likelihood that compensatory engagement becomes poorly controlled and develops into PSMU (19). Thus, optimal-arousal accounts explain why stimulation is sought, compensatory internet use theory explains why social media is selected as a regulatory response, and reinforcement describes how repeated compensatory use may become problematic. Empirical evidence has consistently demonstrated a positive association between boredom proneness and PSMU (31). Accordingly, this integrated pathway supports testing whether occasion-specific elevations in boredom proneness scores prospectively predict higher PSMU at the subsequent wave.

The same integrated account also includes a possible feedback pathway from PSMU to later boredom-proneness scores. Rather than constituting a separate theoretical explanation, this pathway extends the compensatory process by considering whether repeated problematic engagement may prospectively precede greater difficulty maintaining attention, interest, and adequate stimulation. Although boredom proneness is a dispositional construct, longitudinal research in adjacent digital contexts has reported reciprocal associations between boredom proneness and mobile phone addiction and between boredom proneness and problematic online video watching (32, 33). These trait-level findings provide a basis for testing whether PSMU prospectively predicts later boredom proneness scores without equating momentary state boredom with trait boredom proneness. Together, the proposed account conceptualizes the boredom proneness-to-PSMU pathway as compensatory arousal regulation and the PSMU-to-boredom proneness pathway as a possible longitudinal feedback process. The present RI-CLPM tests the longitudinal endpoints of these pathways, but it does not directly test the proposed intervening processes, such as compensatory motives, reinforcement, or attentional change.

Although boredom proneness is a dispositional construct, longitudinal research in adjacent digital contexts suggests that problematic digital engagement may be prospectively associated with later boredom-proneness scores. A two-wave cross-lagged study found reciprocal associations between boredom proneness and mobile phone addiction, with mobile phone addiction more strongly predicting subsequent boredom proneness than the reverse (33). More recent two-wave research likewise reported reciprocal associations between boredom proneness and problematic online video watching (32). Although these studies examined adjacent forms of problematic digital use rather than adolescent PSMU specifically, they provide trait-level longitudinal grounds for testing a possible PSMU-to-boredom-proneness pathway. Because direct longitudinal evidence specific to adolescent PSMU remains limited, the present study tests this reverse pathway rather than assuming it. Accordingly, this evidence also supports separately testing the prospective within-person pathway from PSMU to subsequent boredom-proneness scores. Nonetheless, the reciprocal dynamics between boredom proneness and PSMU among adolescents remain insufficiently explored. Accordingly, this study further hypothesizes a bidirectional predictive relationship between boredom proneness and PSMU.

### The current study

**Figure 1:**
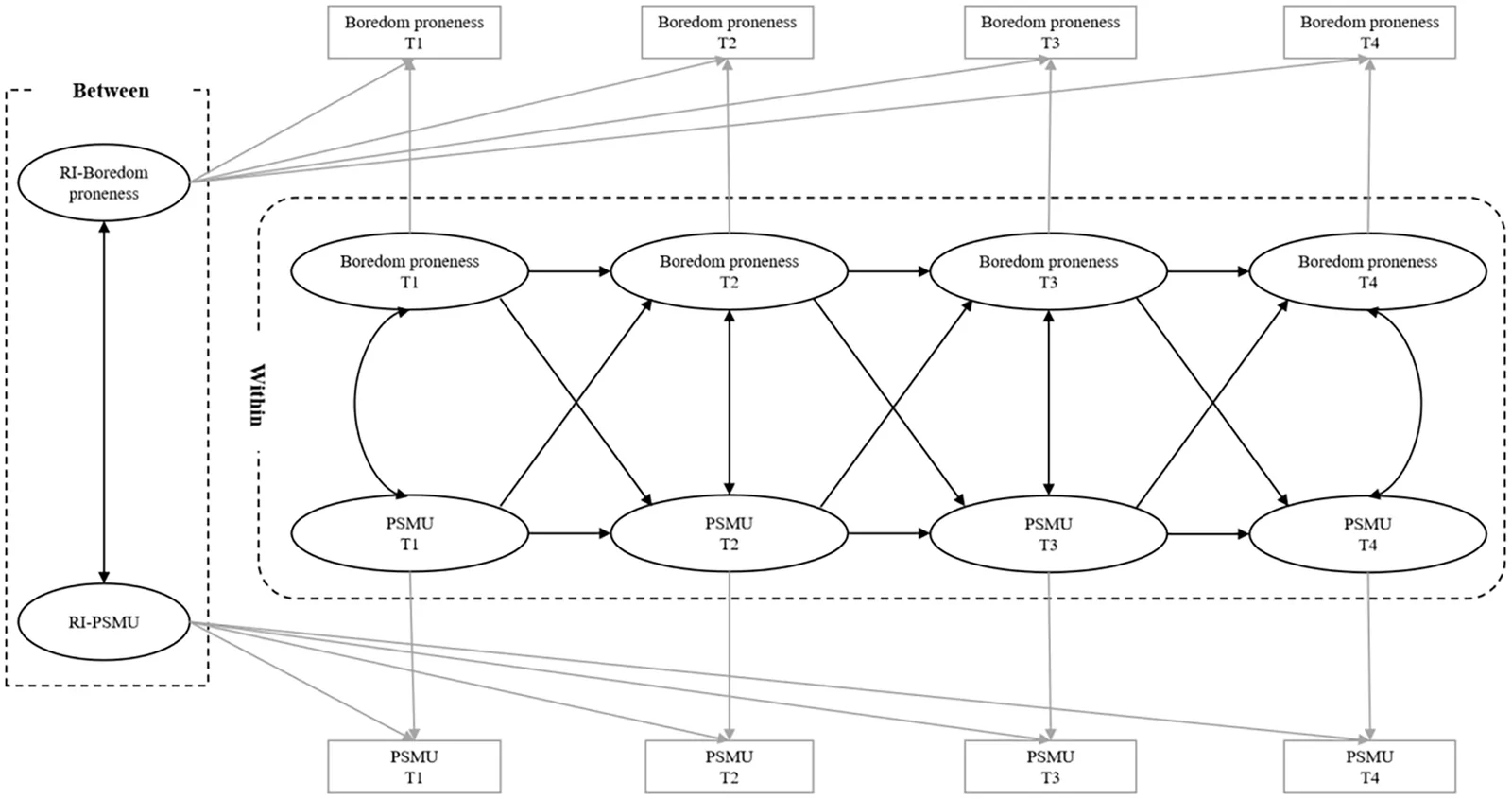
Conceptual RI-CLPM for boredom proneness and PSMU across four waves. Observed variables are represented by squares; latent variables at both the between- and within-person levels are represented by circles; RIs indicate the between-person variance. Correlations between RIs and correlations between the within-person values from the same measurement occasion are specified by black double arrows. Diagonal black arrows represent the cross-lagged paths. Horizontal arrows represent the autoregressive paths. Gender, age, and parents’ educational level are included in the model as control variables but are not shown in the figure for clarity. PSMU, problematic social media use.

Four-wave RI-CLPM designs have already been used to examine problematic digital use in relation to mental health among Chinese college students (34) and FoMO and life satisfaction among Dutch adolescents (15). A recent two-wave adult study further examined metacognitive pathways to PSMU (35). Earlier longitudinal research directly linking boredom proneness to problematic digital engagement mainly examined mobile phone addiction or problematic online video watching, used two-wave conventional cross-lagged models, and sampled university students (32, 33). It therefore remains unclear whether the direct association between trait boredom proneness and PSMU among adolescents reflects stable between-person differences, prospective within-person dynamics, or both. Thus, the novelty of the present study lies not in the four-wave RI-CLPM itself, but in applying it to the direct reciprocal association between trait boredom proneness and PSMU among Chinese adolescents. However, cross-sectional research designs present notable methodological limitations. First, data collected at a single time point cannot establish the temporal order of variables or clarify the direction of causality. Second, such designs fail to disentangle cross-time causal effects from stable trait-like differences across individuals, which may lead to an overestimation of the strength of certain relationships. To overcome these limitations, researchers in psychology and media studies have increasingly adopted the Random Intercept Cross-Lagged Panel Model (RI-CLPM, see **Figure 1**), proposed by Hamaker et al. (21). The RI-CLPM separates stable between-person differences, represented by the random intercepts, from occasion-specific within-person deviations in repeated scores (21). This distinction is particularly relevant for boredom proneness: the random intercept captures stable between-person differences in the dispositional construct, whereas the within-person component captures deviations from an adolescent’s own expected boredom proneness score at a given wave. These deviations should not be interpreted as momentary state boredom or as evidence of definitive enduring personality change. Accordingly, the RI-CLPM provides an appropriate analytical approach for examining temporal associations between boredom proneness and PSMU.

Accordingly, the present study adopted a four-wave longitudinal design spanning 2020 to 2022 to examine the associations between boredom proneness and adolescent PSMU at both the between-person and within-person levels. The RI-CLPM separates stable, time-invariant between-person differences from occasion-specific within-person deviations (21). At the between-person level, the association between the random intercepts indicates whether adolescents who are generally higher in boredom proneness across the study period are also generally higher in PSMU. At the within-person level, the cross-lagged paths indicate whether an occasion-specific deviation in one construct from an adolescent’s own expected level prospectively predicts a deviation in the other construct at the subsequent wave, after accounting for prior within-person deviations. Based on the preceding theoretical and empirical evidence, we proposed the following hypotheses:

H1. At the between-person level, the stable between-person component of boredom proneness will be positively associated with the stable between-person component of PSMU; adolescents who are generally higher in boredom proneness across the study period will also be generally higher in PSMU.

H2. At the within-person level, an occasion-specific elevation in an adolescent’s boredom-proneness score relative to their own expected level at one wave will prospectively predict a higher-than-expected PSMU score at the subsequent wave.

H3. At the within-person level, an occasion-specific elevation in an adolescent’s PSMU score relative to their own expected level at one wave will prospectively predict a higher-than-expected boredom-proneness score at the subsequent wave.

By distinguishing these two levels of analysis, the present study aims to determine whether the association between boredom proneness and PSMU reflects stable between-person co-occurrence, prospective within-person dynamics, or both.

## Methods

### Participants and procedures

At Time 1 (T1, September–October 2020), 2163 students (1137 males and 1026 females, Mage = 14.54 years, SD = 1.49) from two junior high schools and two senior high schools in Guangzhou, China, were invited to participate in this study. All participants were Han Chinese adolescents from southern China. Among these, 1976 adolescents (1033 males and 943 females, retention rate = 91.35%), 1501 adolescents (729 males and 772 females, retention rate = 69.39%), and 1982 adolescents (1037 males and 945 females, retention rate = 91.63%) participated in the assessments at T2 (February–March 2021), T3 (September–October 2021) and T4 (October–November 2022), respectively. The intervals between measurement waves were unequal. Participation was assessed separately at each wave: adolescents who missed an intermediate assessment remained eligible to participate at subsequent waves, and no new participants were recruited after T1. Therefore, the larger sample at T4 reflects the return of baseline participants who had missed one or both intermediate assessments rather than the addition of new participants. All four measurement waves were conducted during the COVID-19 pandemic. Although the surveys were administered during regular class sessions, pandemic-related public-health measures and disruptions to adolescents’ offline routines may have varied across the study period. Individual-level exposure to quarantine, remote learning, mobility restrictions, or perceived social isolation was not assessed. Ethical approval was obtained from the Ethics Committee of Guangzhou University. All procedures involving human participants in this study complied with the ethical standards of the relevant institutional and/or national research committees, as well as the 1964 Declaration of Helsinki and its subsequent amendments or equivalent ethical guidelines.

According to Little’s MCAR test (χ²(111) = 119.07, *p* = 0.283), the missing data followed a completely random pattern. No significant differences were found in boredom proneness (*p* = 0.519) or PSMU (*p* = 0.573) between adolescents who completed all four waves and those who did not. However, gender differences were observed, with boys more likely to drop out than girls (*p* < 0.001). Missing data were handled using the full information maximum likelihood (FIML) approach in Mplus 8.3. Prior to data collection, informed written consent was obtained from both adolescents and their parents, with assurances of voluntary participation and the right to withdraw at any point. Data were collected during regular class sessions at each wave, administered by two trained research assistants who were available to provide clarification as needed. Overall, these procedures suggest that the dataset was unlikely to suffer from attrition bias.

### Measures

#### Boredom proneness

The eight-item Short Boredom Proneness Scale (SBPS), originally developed by Struk et al. (17), was used to assess adolescents’ boredom proneness. A Chinese version has been formally translated and validated among Chinese college students, supporting its unidimensional structure, internal consistency, test–retest reliability, and criterion-related validity (36). Participants rated the items on a 5-point Likert scale ranging from 1 (strongly disagree) to 5 (strongly agree; e.g., “Many things that I have to do are repetitive and monotonous”). The scale demonstrated adequate internal consistency across all four waves. Cronbach’s α coefficients were 0.871, 0.881, 0.915, and 0.902 at T1, T2, T3, and T4, respectively, and McDonald’s ω coefficients were 0.875, 0.885, 0.918, and 0.906, respectively.

#### Problematic social media use (PSMU)

PSMU was assessed using the seven-item adapted C-VAT measure employed by Franchina et al. (4) among Flemish adolescents. The Chinese-language measure has subsequently been used in Chinese samples, supporting its applicability in this cultural context (37). Adolescents rated the items on a 5-point Likert scale ranging from 0 (*never*) to 4 (*always*; e.g., “How frequently do you do your homework poorly because you prefer being on social media?”). The scale demonstrated adequate internal consistency across all four waves. Cronbach’s α coefficients were 0.856, 0.882, 0.890, and 0.887 at T1, T2, T3, and T4, respectively, and McDonald’s ω coefficients were 0.858, 0.882, 0.890, and 0.906, respectively.

#### Covariates

Respondents’ gender was assessed by asking them to indicate whether they were male or female (1 = male, 2 = female). They also reported their father’s and mother’s highest educational attainment (1 = primary school, 2 = middle school or equivalent, 3 = a bachelor’s degree or equivalent, 4 = a master’s degree or above). In addition, respondents’ age at T1 was recorded.

#### Analytic strategy

We used descriptive statistics and bivariate correlation analyses in IBM SPSS 26 to capture the means, standard deviations, and inter-scale correlations of the main study variables. The random-intercept cross-lagged panel model (RI-CLPM) was estimated using Mplus 8.3 to examine the longitudinal associations between boredom proneness and PSMU (see **Figure 1** for the conceptual model). While the traditional cross-lagged panel model does not distinguish between-person and within-person effects, the RI-CLPM can effectively disentangle effects on the within-person level (i.e., autoregressive paths, concurrent paths, cross-lagged paths) from effects on the between-person level (i.e., random intercepts), thereby providing more precise estimates of the associations between boredom proneness and PSMU (21). The autoregressive paths of boredom proneness and PSMU indicate whether an adolescent’s occasion-specific deviation from their own expected score persists to the subsequent wave. The concurrent paths represent the association between deviations in boredom-proneness scores and PSMU on the same measurement occasion (e.g., between boredom T1 and PSMU T1). The cross-lagged paths estimate prospective within-person associations between occasion-specific deviations in the two constructs across adjacent waves. The random intercepts are stable, time-invariant between-person differences in boredom and PSMU. Factor loadings for these random intercepts were fixed to one. Following Mulder and Hamaker’s (38) recommendation, the observed scores of boredom proneness and PSMU at each wave were regressed on gender, age, paternal education, and maternal education. These variables were included as background sociodemographic covariates rather than as a comprehensive set of individual, family, peer, and platform-related determinants of PSMU. Accordingly, the model was not intended to estimate the incremental contribution of boredom proneness relative to all other predictors of PSMU. To align the RI-CLPM parameters with the proposed hypotheses, H1 was evaluated by the association between the random intercepts of boredom proneness and PSMU. H2 was evaluated by the within-person cross-lagged paths from boredom-proneness scores at time T to PSMU at time T + 1, whereas H3 was evaluated by the corresponding cross-lagged paths from PSMU at time T to boredom-proneness scores at time T + 1. To examine potential subgroup heterogeneity, exploratory multigroup RI-CLPMs were additionally conducted by gender (boys, *n* = 1,137; girls, *n* = 1,026) and school stage (junior high school, *n* = 851; senior high school, *n* = 1,312). For each grouping variable, an unconstrained model in which the autoregressive and cross-lagged paths were freely estimated across groups was compared with a constrained model in which the corresponding lagged regression coefficients were set equal across groups. Chi-square difference tests were used to evaluate overall subgroup differences. When the overall model comparison was significant, path-specific parameter-difference tests were conducted to identify the source of the difference.

## Results

### Preliminary analyses

As **Table 1** shows, the means, standard deviations, and bivariate correlations for boredom proneness and PSMU are presented.

**Table 1:** Descriptive statistics and correlations of study variables.

Variables	1	2	3	4	5	6	7	8	9	10	11	12
1. Age	–											
2. Gender	0.056^**^	–										
3. Fathers’ education level	-0.396^***^	-0.043^*^	–									
4. Mothers’ education level	-0.411^***^	-0.050^*^	0.700^***^	–								
5. Boredom proneness T1	0.239^***^	0.068^**^	-0.174^***^	-0.182^***^								
6. Boredom proneness T2	0.228^***^	0.053^*^	-0.184^***^	-0.213^***^	0.628^***^	–						
7. Boredom proneness T3	0.193^***^	0.067^**^	-0.119^***^	-0.140^***^	0.599^***^	0.630^***^	–					
8. Boredom proneness T4	0.100^***^	0.097^***^	-0.123^***^	-0.160^***^	0.480^***^	0.521^***^	0.586^***^	–				
9. Problematic social media use T1	0.330^***^	0.184^***^	-0.213^***^	-0.220^***^	0.455^***^	0.378^***^	0.365^***^	0.275^***^	–			
10. Problematic social media use T2	0.310^***^	0.157^***^	-0.179^***^	-0.177^***^	0.391^***^	0.462^***^	0.412^***^	0.325^***^	0.625^***^	–		
11. Problematic social media use T3	0.298^***^	0.237^***^	-0.171^***^	-0.199^***^	0.345^***^	0.379^***^	0.414^***^	0.341^***^	0.591^***^	0.663^***^	–	
12. Problematic social media use T4	0.188^***^	0.152^***^	-0.137^***^	-0.150^***^	0.319^***^	0.316^***^	0.306^***^	0.388^***^	0.475^***^	0.508^***^	0.584^***^	–
*M*	14.54	1.47	2.52	2.44	2.33	2.43	2.38	2.31	1.29	1.30	1.27	1.28
*SD*	1.49	0.50	0.74	0.77	0.85	0.82	0.81	0.87	0.86	0.89	0.88	0.88

^*^p <0.05, ^**^p <0.01, ^***^p <0.001.

Gender was coded as 1 = male, 2 = female. parental educational levels: 1 = primary school, 2 = middle school or equivalent, 3 = a bachelor’s degree or equivalent, 4 = a master’s degree or above. T1 = Time 1, T2 = Time 2, T3 = Time 3, T4 = Time 4.

### Model testing

As a preliminary step, measurement invariance was tested to ensure that factor loadings remained consistent across time points. Establishing at least metric invariance (i.e., weak factorial invariance) is essential for valid longitudinal comparisons. In this study, the same factor structure and loadings were confirmed across all four waves, indicating longitudinal metric invariance (see **Table 2**). The changes in model fit indices (ΔCFI, ΔTLI ≤ 0.01; ΔRMSEA ≤ 0.015) met the recommended thresholds proposed by Cheung and Rensvold (39), supporting the equivalence of measurement over time.

**Table 2:** Measurement scale invariance over time.

Model	*χ2*/*df*	CFI	TLI	RMSEA (90% CI)	SRMR	ΔCFI	ΔTLI	ΔRMSEA
Boredom proneness								
1. Configural Invariance	5.922	0.944	0.933	0.048 [0.046, 0.050]	0.034	–	–	–
2. Weak Factorial Invariance	5.785	0.942	0.935	0.047 [0.045, 0.049]	0.037	0.002	-0.002	0.001
3. Strong Factorial Invariance	6.426	0.932	0.926	0.050 [0.048, 0.052]	0.039	0.010	0.009	-0.003
Problematic social media use								
1. Configural Invariance	8.180	0.934	0.920	0.058 [0.056, 0.060]	0.043	–	–	–
2. Weak Factorial Invariance	8.327	0.928	0.919	0.058 [0.056, 0.060]	0.047	0.006	0.001	0.000
3. Strong Factorial Invariance	8.997	0.918	0.911	0.061 [0.059, 0.063]	0.049	0.010	0.008	-0.003

### RI-CLPM

**Figure 2:**
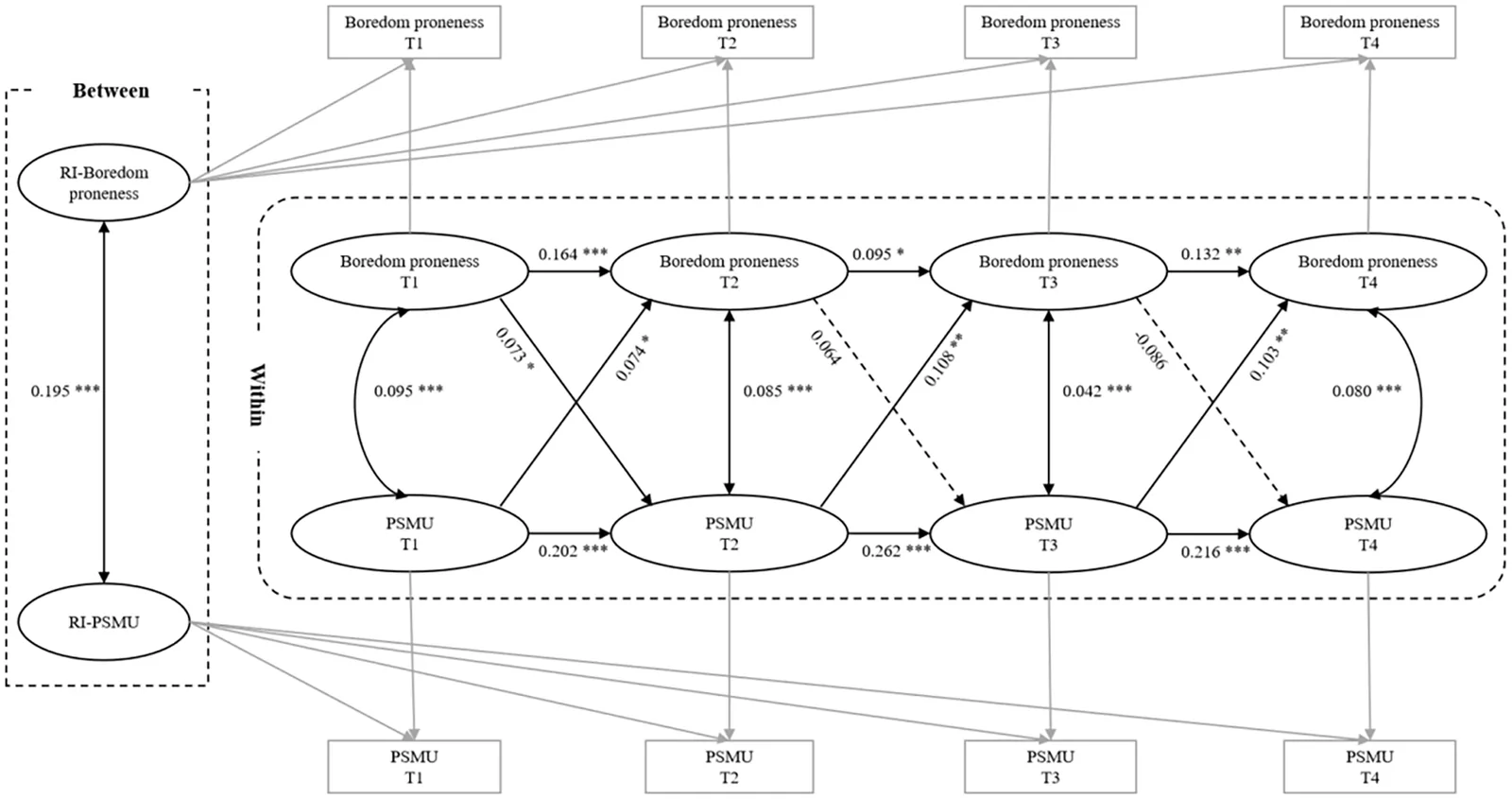
The RI-CLPM for boredom proneness and PSMU. Unstandardized solutions are reported. ^*^p < 0.05, ^**^p < 0.01, ^***^p < 0.001.

The RI-CLPM showed an acceptable model fit: χ²(37) = 315.569, CFI = 0.963, TLI = 0.940, RMSEA = 0.059, SRMR = 0.087. As shown in **Figure 2**, within-person deviations in boredom proneness and PSMU showed significant autoregressive effects across adjacent waves. The autoregressive paths revealed that within-person deviations in boredom proneness at T2 were predicted by deviations in boredom proneness at T1 (B = 0.164, *p* < 0.001), boredom proneness at T3 was predicted by deviations in boredom proneness at T2 (B = 0.095, *p* < 0.05), boredom proneness at T4 was predicted by deviations in boredom proneness at T3 (B = 0.132, *p* < 0.01), PSMU at T2 was predicted by deviations in PSMU at T1 (B = 0.202, *p* < 0.001), PSMU at T3 was predicted by deviations in PSMU at T2 (B = 0.262, *p* < 0.001), and PSMU at T4 was predicted by deviations in PSMU at T3 (B = 0.216, *p* < 0.001). Examining the cross-lagged paths, higher PSMU at time T was associated with higher levels of boredom proneness at T + 1 (B = 0.074, 0.108 and 0.103; *p* < 0.05, 0.01 and 0.01), and higher boredom proneness at time T1 was associated with higher levels of PSMU at T2 (B = 0.073, *p* < 0.05). However, boredom proneness at time T2 could not predict PSMU at T3, and boredom proneness at time T3 could not predict PSMU at T4 (B = 0.046 and -0.086, *p* > 0.05 and > 0.05). A significant positive relation between PSMU and boredom proneness was found (B = 0.195, *p* < 0.001), which means that adolescents with higher overall (between-person) levels of PSMU also showed higher overall levels of boredom proneness across the study period. Thus, despite time-specific fluctuations and associations, adolescents’ PSMU and boredom proneness were positively related at the between-person level, implying that adolescents who reported higher average PSMU were also more likely to report higher average boredom proneness. In addition, significant within-person concurrent associations were observed at each wave, demonstrating that higher-than-expected levels of PSMU were related to correspondingly higher-than-expected levels of boredom proneness at the same measurement occasion (B = 0.095, 0.085, 0.042, and 0.080; *p* < 0.001, *p* < 0.001, *p* < 0.001, *p* < 0.001). Accordingly, H1 was supported because the stable between-person components of boredom proneness and PSMU were positively associated. H2 received partial support, as the within-person path from boredom-proneness scores to subsequent PSMU was significant only from T1 to T2 but not across the two later intervals. H3 was supported, as within-person elevations in PSMU consistently predicted subsequent elevations in boredom-proneness scores across all three adjacent intervals.

### Subgroup analyses

Exploratory multigroup RI-CLPMs were conducted by gender and school stage. For gender, the unconstrained model showed a good fit, χ²(18) = 45.347, CFI = 0.996, TLI = 0.988, RMSEA = 0.037, and SRMR = 0.022. Constraining the corresponding autoregressive and cross-lagged paths to equality across boys and girls did not significantly worsen model fit, χ²(30) = 62.375, CFI = 0.995, TLI = 0.991, RMSEA = 0.032, and SRMR = 0.025; Δχ²(12) = 17.028, *p* = 0.149. Thus, no significant overall gender difference was found in the lagged associations.

For school stage, the unconstrained model showed a good fit, χ²(18) = 43.803, CFI = 0.996, TLI = 0.987, RMSEA = 0.036, and SRMR = 0.023. Constraining the corresponding autoregressive and cross-lagged paths to equality across junior- and senior-high-school students resulted in a significant deterioration in model fit, χ²(30) = 67.809, CFI = 0.994, TLI = 0.989, RMSEA = 0.034, and SRMR = 0.029; Δχ²(12) = 24.006, *p* = 0.020. However, path-specific parameter-difference tests indicated that none of the six focal cross-lagged paths between boredom proneness and PSMU differed significantly across school stages (all *ps* ≥ 0.082). Among the tested lagged parameters, the only significant difference was observed for the PSMU T3-to-T4 autoregressive path, ΔB = 0.190, *p* = 0.010. Specifically, this autoregressive association was stronger among junior-high-school students (B = 0.361, *p* < 0.001) than among senior-high-school students (B = 0.171, *p* < 0.001). Thus, the overall school-stage difference in the lagged model appeared to be attributable to differences in the temporal stability of PSMU rather than differences in the focal reciprocal associations between boredom proneness and PSMU.

## Discussion

Overall, the findings supported H1 and H3, whereas H2 received only interval-specific support. Thus, the association between boredom proneness and PSMU involved both stable between-person co-occurrence and asymmetric prospective within-person associations. At the between-person level, boredom proneness was positively associated with PSMU, suggesting that adolescents with higher average levels of boredom proneness tended to report higher average levels of problematic social media use. The findings indicated that boredom proneness and PSMU exhibited significant concurrent (within-wave) associations across four measurement waves (T1–T4). At the between-person level, a significant positive association was observed between the two constructs, with higher overall boredom proneness being associated with higher levels of PSMU. This pattern is consistent with the compensatory internet use theory (27), which posits that individuals engage in online activities to compensate for unmet psychological or social needs in offline contexts. Adolescents characterized by elevated boredom proneness often display attenuated sensitivity to real-world stimuli, rendering them more inclined to seek emotional gratification or social connectedness through social media, thereby fostering habitual and compensatory usage patterns.

Consistent with prior research, the findings identify boredom proneness as one theoretically relevant dispositional correlate of dysregulated digital engagement (18, 24). In other words, individuals with higher levels of boredom proneness may be more inclined to engage in compulsive social media behaviors, such as prolonged scrolling through content or repeatedly seeking opportunities for online interaction with peers. Consequently, social media platforms may function as a maladaptive coping mechanism for managing chronic boredom-related experiences, which in turn may increase the risk of developing PSMU.

At the between-person level, the robust association between boredom proneness and PSMU is consistent with prior empirical evidence (40), indicating that individuals with elevated susceptibility to boredom are more likely to exhibit maladaptive patterns of digital media use. However, within-person analyses indicated an asymmetrical pattern of cross-lagged associations. Specifically, boredom proneness was associated with subsequent increases in PSMU only at the first temporal lag (T1→T2), whereas this association was not observed across later lags, suggesting that its prospective association with PSMU was limited to the first observed interval rather than remaining stable across the study period. Conversely, PSMU demonstrated a consistent association with subsequent increases in boredom proneness across all temporal lags, a pattern broadly consistent with trait-level longitudinal findings involving mobile phone addiction and problematic online video watching (32, 33). Tam and Inzlicht’s (41) experimental finding that rapid switching among online videos increased immediate state boredom may suggest a possible short-term process involving reduced attention and engagement. However, this state-level evidence does not demonstrate changes in trait boredom proneness. Likewise, the within-person component in the present RI-CLPM represents occasion-specific deviations in repeated boredom-proneness scores rather than momentary state boredom or definitive enduring personality change. The processes through which repeated digital experiences may become associated with longer-term variation in boredom proneness therefore remain to be tested.

One plausible explanation for this asymmetry concerns the different temporal roles of a dispositional vulnerability and a behavioral pattern. Because the RI-CLPM separates stable between-person differences from within-person deviations, the boredom proneness-to-PSMU paths indicate whether an occasion-specific elevation in an adolescent’s boredom-proneness score above their own expected level predicts subsequent PSMU (21). Such elevations may activate compensatory social media use only under particular conditions, which may explain why this pathway emerged from T1 to T2 but not consistently across all intervals. The unequal lag lengths provide an additional temporal explanation: T1–T2 was the shortest interval, suggesting that the boredom proneness-to-PSMU association may operate over a relatively proximal timescale and become less detectable across longer intervals. By contrast, PSMU represents an enacted and potentially recurrent behavioral pattern. Repeated problematic engagement may therefore show a more persistent prospective association with later difficulties in sustaining attention and interest, consistent with the significant PSMU-to-boredom proneness paths across all three intervals. Nevertheless, because the proposed intervening processes were not measured, the intervals were unequal, and the reciprocal coefficients were not formally compared, the findings should be interpreted as asymmetric temporal precedence rather than evidence that PSMU has a definitively stronger causal effect than boredom proneness. The COVID-19 pandemic provides an additional historical context for interpreting these findings. Disruptions to face-to-face interaction, school routines, and offline recreational activities may have increased adolescents’ experiences of understimulation or social isolation while also increasing reliance on digital communication. Research conducted during the pandemic found that loneliness was associated with problematic mobile phone use among Chinese adolescents through escape motivation (42), while boredom proneness was implicated in problematic smartphone use among Chinese adolescents during the same period (11). These findings suggest that pandemic-related disruption could plausibly have influenced both boredom proneness and problematic digital engagement. However, the present study did not assess participants’ actual exposure to restrictions, remote learning, or social isolation. We therefore cannot determine whether the pandemic strengthened the association between boredom proneness and PSMU or contributed to the interval-specific T1-to-T2 pathway. The findings should be interpreted within this historical context and replicated in post-pandemic cohorts.

This interpretation is consistent with theoretical accounts that conceptualize boredom experiences as signals of insufficient stimulation or meaning; the present study extends these accounts by examining the dispositional susceptibility to experiencing such states (43, 44).

The present study extends prior research in three respects. First, whereas previous longitudinal cross-lagged studies have examined problematic digital use in relation to mental health, FoMO, life satisfaction, or other metacognitive processes, the present study focuses on the direct reciprocal association between trait boredom proneness and PSMU during adolescence (15, 34, 35). Second, it extends two-wave conventional cross-lagged findings from university samples by separating stable between-person co-occurrence from prospective within-person dynamics across four waves (32, 33). Third, the findings show that the two levels yield different conclusions: boredom proneness and PSMU were positively associated at the between-person level, whereas the within-person association was asymmetric, with PSMU consistently preceding higher boredom-proneness scores and the reverse pathway emerging only from T1 to T2.

These findings should also be interpreted within the platform–family–school ecology of Chinese adolescents. Multifunctional communication platforms such as WeChat and QQ coexist with algorithm-driven short-video platforms such as Douyin, while study-related stress, difficulties in family and school relationships, parental phubbing, and parental mediation have been linked to problematic digital engagement among Chinese youth (9–11). This ecology may help contextualize why social media represents a readily accessible source of stimulation and coping for Chinese adolescents. However, these contextual factors were not directly measured, and the observed pathways should not be interpreted as uniquely Chinese.

## Implications

This study advances understanding of adolescent PSMU by adopting the RI-CLPM to disentangle within-person and between-person processes. The findings suggest a differentiated, two-level approach to prevention. At the between-person level, the positive association between boredom proneness and PSMU indicates that adolescents who consistently report high levels of either construct may benefit from assessment of the other. At the within-person level, the consistent PSMU-to-boredom-proneness paths suggest that increases in PSMU relative to an adolescent’s own usual level may serve as an early warning signal. Schools and families could therefore use repeated brief check-ins, rather than relying only on one-time assessments or group averages, to identify sustained increases in problematic use and provide timely support for planned social media use and meaningful offline engagement. In contrast, because boredom proneness predicted PSMU only from T1 to T2, boredom-focused support should be applied as a complementary and time-sensitive strategy during periods of elevated boredom-proneness scores, rather than assumed to prevent PSMU uniformly across time. Thus, the asymmetric pattern points to PSMU management as the more consistently indicated prospective target, while boredom coping remains a conditional target. Such support may be particularly relevant during periods of disrupted schooling or restricted offline social and recreational opportunities, when adolescents may rely more heavily on digital stimulation; however, the present study does not establish that pandemic-related restrictions intensified the observed pathways. Because the study was observational and the reciprocal coefficients were not formally compared, these implications should be tested in intervention studies rather than interpreted as established causal recommendations.

## Limitations and future research

This study has several limitations that warrant consideration in future research. First, the data were collected through adolescent self-reports, which may be vulnerable to social desirability bias or recall bias when measuring subjective constructs such as boredom proneness and PSMU (45). To enhance the reliability and validity of findings, future studies could incorporate information from multiple sources (e.g., parents, teachers, and peers) to triangulate key variables (46). Second, although the four-wave longitudinal design provided preliminary evidence for the bidirectional associations between boredom proneness and PSMU, the relatively wide spacing between measurement waves may have limited the ability to capture short-term dynamic fluctuations. Future research could adopt intensive longitudinal designs, such as the Experience Sampling Method (ESM), which offer higher temporal resolution for tracking moment-to-moment changes and causal directions (47). The present study assessed trait boredom proneness rather than momentary state boredom. Accordingly, the within-person component of the RI-CLPM represents occasion-specific deviations in repeated boredom-proneness scores and should not be interpreted as a direct measure of state boredom. Although experimental studies of state boredom may suggest possible short-term processes through which digital media use affects attention and engagement, the present data cannot determine whether repeated episodes of state boredom accumulate into longer-term changes in boredom proneness. Future research should combine intensive assessments of state boredom, such as experience sampling, with multiwave measures of boredom proneness and PSMU to directly examine the potential links between state-level experiences and dispositional change. In addition, all four waves were conducted during the COVID-19 pandemic, but the study did not measure participants’ exposure to quarantine, remote learning, mobility restrictions, pandemic-related stress, or perceived social isolation. These unmeasured contextual conditions may have influenced levels of both boredom proneness and PSMU as well as their longitudinal associations. Consequently, the findings may partly reflect the specific historical circumstances of the pandemic and should be replicated in post-pandemic samples. Third, while the RI-CLPM effectively distinguishes between within-person and between-person effects, the bidirectional relationships observed in the model may not generalize across all adolescent groups. Age, gender, or other contextual factors may moderate the within-person links between boredom proneness and PSMU (48). Future studies could extend the RI-CLPM by incorporating interaction terms or multigroup structures to explore heterogeneity in causal pathways (49). Fourth, the present RI-CLPM intentionally focused on the reciprocal association between boredom proneness and PSMU and did not include other individual, family, peer, or platform-related determinants of PSMU. Although the model separates stable between-person differences from within-person deviations, unmeasured time-varying factors may still have influenced the estimated within-person paths. Therefore, the findings should not be interpreted as showing that boredom proneness is the sole or strongest determinant of PSMU or that it has a unique effect after comprehensive adjustment. Future research should use multivariate RI-CLPMs or related longitudinal models to compare boredom proneness with other theoretically relevant predictors and examine whether the focal associations remain after accounting for them. Fifth, participants ranged from 12 to 19 years, covering multiple phases of adolescence. Although the exploratory multigroup analyses examined differences by gender and school stage, school stage represents only a broad indicator of adolescent development, and continuous age moderation was not tested. Moreover, the isolated school-stage difference in the PSMU T3-to-T4 autoregressive path emerged from exploratory path-specific comparisons and should be replicated before firm developmental conclusions are drawn. Future research should examine developmental heterogeneity using more fine-grained age classifications or moderated RI-CLPMs. In addition, all participants were Han Chinese adolescents recruited from four schools in Guangzhou; therefore, the findings should be interpreted within this regional and cultural context and should not be generalized to all Chinese adolescents or other cultural groups without replication.

## Conclusion

1. At the between-person level, boredom proneness was positively associated with PSMU, indicating stable co-occurrence between the two constructs.
2. At the within-person level, an asymmetric bidirectional association was observed: boredom proneness significantly predicted subsequent PSMU only from T1 to T2, whereas PSMU consistently predicted subsequent boredom proneness across three consecutive intervals. This suggests that, while boredom proneness may initially drive adolescents to seek stimulation through social media, higher-than-usual PSMU was consistently followed by higher-than-usual boredom-proneness scores, indicating a possible asymmetric prospective feedback pattern. This finding should not be interpreted as evidence that PSMU acutely induces state boredom or definitively changes an enduring personality trait.
3. Both boredom proneness and PSMU showed significant autoregressive effects, indicating a degree of temporal stability in these constructs.

## Data Availability

The raw data supporting the conclusions of this article will be made available by the authors, without undue reservation.
